# Predicting the Treatment Outcomes of Antidepressants Using a Deep Neural Network of Deep Learning in Drug-Naïve Major Depressive Patients

**DOI:** 10.3390/jpm12050693

**Published:** 2022-04-26

**Authors:** Ping-Lin Tsai, Hui Hua Chang, Po See Chen

**Affiliations:** 1 Institute of Clinical Pharmacy and Pharmaceutical Sciences, College of Medicine, National Cheng Kung University, Tainan 701, Taiwan; 3.1415926legotsai@gmail.com; 2 School of Pharmacy, College of Medicine, National Cheng Kung University, Tainan 701, Taiwan; 3 Department of Pharmacy, National Cheng Kung University Hospital, College of Medicine, National Cheng Kung University, Tainan 701, Taiwan; 4 Department of Pharmacy, National Cheng Kung University Hospital, Dou-Liou Branch, Yunlin 640, Taiwan; 5 Department of Psychiatry, National Cheng Kung University Hospital, College of Medicine, National Cheng Kung University, Tainan 701, Taiwan; chenps@mail.ncku.edu.tw; 6 Institute of Behavioral Medicine, College of Medicine, National Cheng Kung University, Tainan 701, Taiwan

**Keywords:** major depressive disorder, antidepressant, deep neural network, deep learning, polymorphisms

## Abstract

Predicting the treatment response to antidepressants by pretreatment features would be useful, as up to 70–90% of patients with major depressive disorder (MDD) do not respond to treatment as expected. Therefore, we aim to establish a deep neural network (DNN) model of deep learning to predict the treatment outcomes of antidepressants in drug-naïve and first-diagnosis MDD patients during severe depressive stage using different domains of signature profiles of clinical features, peripheral biochemistry, psychosocial factors, and genetic polymorphisms. The multilayer feedforward neural network containing two hidden layers was applied to build models with tenfold cross-validation. The areas under the curve (AUC) of the receiver operating characteristic curves were used to evaluate the performance of the models. The results demonstrated that the AUCs of the model ranged between 0.7 and 0.8 using a combination of different domains of categorical variables. Moreover, models using the extracted variables demonstrated better performance, and the best performing model was characterized by an AUC of 0.825, using the levels of cortisol and oxytocin, scales of social support and quality of life, and polymorphisms of the *OXTR* gene. A complex interactions model developed through DNN could be useful at the clinical level for predicting the individualized outcomes of antidepressants.

## 1. Introduction

Depression is one of the most common mental illnesses in the world. According to statistical data from the WHO, in 2021, approximately 280 million people worldwide were suffering from depression, which is one of the main causes of disability and a health insurance burden [1]. At its worst, major depressive disorder (MDD) can lead to suicide, impair psychosocial function, and increase the risk of comorbidities, such as cardiovascular disease and obesity [2]. At present, the treatment of MDD is mainly based on antidepressant medication. The main antidepressants currently in use are selective serotonin reuptake inhibitors (SSRIs) and norepinephrine and serotonin reuptake inhibitors (SNRIs). However, only 11–30% of patients treated with antidepressants can achieve a complete remission of their disease, and approximately 50% of patients will not respond at all to the drugs. In addition, the efficacy of antidepressants usually only manifests after receiving medication for 4–8 weeks [3]. Moreover, the treatment response to antidepressants in MDD patients shows individual differences [4]. Therefore, it is vital to develop a helpful approach to predict the efficacy of antidepressants and to decrease the overall burden on health care rather than applying a trial-and-error approach.

Previous studies have demonstrated that certain factors and biomarkers, such as clinical features, psychosocial factors and genetic markers, are associated with the effectiveness of antidepressants [5,6]. Choi et al. reported that inflammation markers such as high-sensitivity C-reactive protein (hsCRP) and life stressors might be useful predictors for short- and long-term treatment responses [7]. In addition, metabolic indices such as blood sugar and insulin levels are suggested to be biomarkers and to play important roles in the psychopathology of MDD and its treatment outcome [2,8,9], as MDD patients have a high risk of comorbid type 2 diabetes, obesity, and metabolic syndrome [10]. Furthermore, genetic polymorphisms contributing to the treatment response to antidepressants have been identified [11,12,13]. Three large genome-wide association studies (GENDEP [11], STAR*D [12], and MARS [13]) have demonstrated an association between genetic variants across the whole genome and the effectiveness of antidepressants, but the small effect size involved in the antidepressant effect limits the clinical application of genetic biomarkers [14]. Taken together, although there are some known predictors associated with MDD and with the treatment response to antidepressants, establishing a predictive model is necessary to tailor the treatment outcomes of individual MDD patients [15].

Recently, personalized medication based on pharmacogenetic data has been proposed to improve the effectiveness of antidepressant treatments in patients with MDD [16]. However, the complexity of the regulation of gene transcription and its interactions with environmental factors means that straightforward translation of individual genetic information into tailored treatment is unlikely. When data from genetic factors, environmental factors, and biomarkers are used in combination, they may lead to the development of useful personalized antidepressant treatment approaches [17,18,19]. Previous studies have demonstrated the predictability of the antidepressant response by applying machine learning strategies, and they suggested that a multivariate approach combining genetic variants and clinical variables could improve the prediction of the antidepressant treatment response [17,18,19]. Among the various machine learning techniques, deep learning demonstrated superior performance in situations with complex data profiles and it has been widely applied in the field of mental illness [20]. Utilizing deep learning can predict the optimal treatment response by identifying potential influencing factors, including demographic and genetic profiles [21]. However, due to the limitations of study designs, medication use, lack of psychosocial factors, and different genetic backgrounds, a predictive model of the antidepressant response still needs to be established for Taiwanese MDD patients. Therefore, we aimed to establish a model to predict the treatment outcomes of antidepressants in drug-naïve and first-diagnosis MDD patients at the severe depressive stage. Furthermore, we aimed to maximize the prediction rate of treatment outcomes of antidepressants using combination profiles of clinical features, peripheral biochemistry, scores on questionnaires evaluating psychosocial factors (quality of life, social support, and recent life events), and genetic variants in these MDD patients.

## 2. Materials and Methods

### 2.1. Subjects

The Institutional Review Board for the Protection of Human Subjects at National Cheng Kung University Hospital approved the research protocol of this study (IRB No. B-ER-108-058). All participants were recruited from outpatient settings at the National Cheng Kung University Hospital (NCKUH) and provided written informed consent regarding their willingness to participate in the research. All MDD patients were diagnosed by an attending psychiatrist and met the criteria for major depressive disorder according to the Diagnostic and Statistical Manual of Mental Disorders, Fourth Edition, Text Revision (DSM-IV-TR). The Chinese version of the Mini International Neuropsychiatry Interview (MINI) was used to determine the diagnosis and confirm the past medical history. The MDD patients also met the following inclusion criteria: (i) 18 to 65 years of age and (ii) a 17-item Hamilton Depression Rating Scale (HDRS) score greater than 15 at the time of study entry. The exclusion criteria were as follows: (i) suffering from a serious suicide tendency; (ii) severe comorbid psychiatric disease such as schizophrenia, bipolar disorder, etc.; (iii) a DSM-IV diagnosis of substance or alcohol abuse within the past year; (iv) severe comorbid physical illness such as cardiovascular, liver, kidney, respiratory system, endocrine, nervous system disease etc.; (v) patients who were pregnant or planned to become pregnant; (vi) having previously taken any category of antidepressant.

The enrolled 70 drug-naïve MDD patients (Supplementary Figure S1) all met the diagnostic criteria of MDD via the DSM-IV-TR criteria at the time of study entry and the HDRS scores >15, as described previously [22]. In the current study, these MDD patients were also diagnosed for the first time. All of the MDD patients included in this study had never received antidepressant treatment prior to enrollment. In addition, the characteristics of MDD patients between enrolled and not enrolled were shown in the Supplementary Tables S1–S3, and they were not significantly different between the groups. After entering the study, they were randomly assigned to either the fluoxetine or the venlafaxine treatment group and treated for 6 weeks. The initial dose of fluoxetine was 20 mg once daily, which could be increased by 20 mg in divided doses to a maximal daily dose of 80 mg. The initial dose of venlafaxine was 37.5 mg once daily for 4 days, titrated to 75 mg once daily, which could be increased by 75 mg in divided doses to a maximal daily dose of 225 mg. The dose of the antidepressant was titrated according to the patient’s disease severity by an attending psychiatrist. Lorazepam was the only allowed concomitant drug, to a maximal daily dose of 6 mg.

All MDD patients were evaluated at the start of the study and then after 2, 4, and 6 weeks using the HDRS, which was administered by a senior attending psychiatrist. The same rater administered the scale at admission and during the subsequent weeks for each patient. Remission of disease was defined as an HDRS score <8 after 6 weeks of treatment.

Additionally, all of the patients had their body mass index (BMI) measured at the start of the study. BMI was calculated as weight (kg) divided by height squared (m^2^), and waist circumference was measured at the level midway between the lateral lower rib margin and the superior anterior iliac crest.

### 2.2. Measurements of Peripheral Biochemistry and Genotyping

Fasting blood samples were collected between 8:00 am and 10:00 am. Ten milliliters of whole blood was withdrawn from the antecubital vein of each patient. Plasma or serum samples, which were isolated from whole blood after centrifugation at 3000× *g* for 15 min at 4 °C, were immediately stored at –80 °C.

#### 2.2.1. Blood Lipid and Sugar Profile

All blood profiles were measured at the laboratory of the Pathology Research Center at NCKU Hospital. Blood lipid profiles, including fasting total cholesterol, high-density lipoprotein (HDL), and triglyceride (TG) concentrations, were detected by enzymatic methods. Low-density lipoprotein (LDL) was calculated by using the Friedewald formula. Fasting plasma glucose values were determined using the glucose oxidase method (Synchron CX3, Beckman, Brea, CA, USA). The HbA1c value was measured using the automated boronate affinity high-performance liquid chromatography method (CLC385; Primus Corp., Kansas City, MO, USA). The fasting serum insulin concentration was measured using a solid-phase radioimmunoassay method (Diagnostic Products Corporation, Los Angeles, CA, USA). The insulin resistance index, which indicated the homeostasis model assessment-estimated insulin resistance (HOMA-IR), was calculated as fasting serum insulin value (μIU/mL) × fasting plasma glucose value (mg/dL)/405 [23]. The homeostasis model assessment for pancreatic β-cell function (HOMA-β) was calculated as 360 × fasting serum insulin value (μIU/mL)/(fasting plasma glucose value (mg/dL) − 63) [23].

#### 2.2.2. Leptin

The fasting plasma leptin level was measured using an ELISA method (Linco Research, St. Louis, MO, USA). The limit of detection was 0.5 ng/mL, and the intra- and interassay coefficients of variation were 7% and 9%, respectively.

#### 2.2.3. C-Reactive Protein

The plasma hsCRP level was determined by an enzyme-linked immunosorbent assay (ELISA) with a human CRP Instant ELISA kit (Bender MedSystem GmbH, Vienna, Austria) following the manufacturer’s instructions. The limit of detection was 3 pg/mL, and the intra- and interassay coefficients of variation (CVs) were 6.9% and 13.1%, respectively.

#### 2.2.4. Oxytocin

The oxytocin immunoreactivity level was quantified in duplicate using a commercial oxytocin ELISA kit (ELISA Kit for oxytocin, USCN Life Science, Houston, TX, USA). The detectable range for this assay was 12.35–1000 pg/mL. The intra-assay coefficient of variation (CV) was 10%, and the interassay CV was 12%. The minimum detectable dose of oxytocin was typically less than 4.87 pg/mL. There was no significant cross-reactivity or interference between oxytocin and the analogs observed. We validated the assay by taking a pool of 10 plasma samples from our subjects and spiking it with a series of oxytocin levels in the physiological range (dilutions from 2–50 pg/mL). The assay accurately reported the increments in the spiked plasma samples (R^2^ = 0.998).

#### 2.2.5. SNP Determination and Genotyping

Genomic DNA was extracted from each blood sample using a QIAamp DNA blood kit (Qiagen, Hilden, Germany) according to the manufacturer’s instructions. The quality of the extracted genomic DNA was checked by agarose gel electrophoresis analysis. The DNA was stored at −80 °C until use. The single nucleotide polymorphisms (SNPs) of the genes selected according to our previous studies (including *BDNF* rs6265, *GNB3* rs5443, *HTR2A* rs6313, *HTR1A* rs6295, *IL1B* rs16944, *TPH1* rs1800532, *SLC6A4* rs25533, and *OXTR* rs53576) [24,25,26,27]. They were analyzed using commercially available TaqMan SNP Genotyping Assays (Applied Biosystems, Foster City, CA, USA) according to the manufacturer’s instructions, and amplification and dissociation were carried out using an ABI 7900HT Fast Real-Time PCR System (Applied Biosystems). The PCR system automatically calculated the negative derivative of the change in fluorescence. The SNP genotype of each tested sample was determined using STEPONE software (Applied Biosystems, Foster City, CA, USA) and confirmed manually. In cases of disagreement, the analysis was repeated.

### 2.3. Questionnaires

#### 2.3.1. World Health Organization Quality of Life (WHOQoL)

The Taiwanese version of the World Health Organization Quality of Life-BREF (WHOQoL-BREF) was used to measure the overall and specific quality of life of all subjects [28]. This questionnaire consists of 28 items in four domains: physical, psychological, social relations, and environment. The reliability and validity of the Taiwanese version of the WHOQoL-BREF were tested. The test–retest reliability coefficient at intervals of 2 to 4 weeks ranged from 0.76 to 0.80 at the domain level. The internal consistency (Cronbach’s alpha) coefficients were in the range of 0.70 to 0.77 for the four domains, and the content validity coefficients were in the range of 0.53 to 0.78 for the item-domain correlations.

#### 2.3.2. Social Support Scale

The social support scale is a 40-item self-report questionnaire that measures perceived and received social support in routine or crisis conditions. It includes four subscales: (i) perceived crisis support (PCS); (ii) perceived routine support (PRS); (iii) received crisis support (RCS); and (iv) received routine support (RRS). The correlations among the four subscales are greater than 0.43 [29].

#### 2.3.3. Life Event Scale

The Recent Life Changes Questionnaire (RLCQ) was developed and modified from the Schedule of Recent Experience (SRE), which was used to collect information concerning the subjects’ recent life changes [30]. In this study, we used the Taiwanese version of the life event scale (LES), which contains 39 items regarding representative life change events in the past 12 months, and the level of perceived stress brought about by recent life-changing events was recorded [31].

### 2.4. Cognitive Function

#### 2.4.1. Finger-Tapping Test (FTT)

A broad range of cognitive deficits have been found in MDD patients, among which motor function, attention, and executive deficits associated with frontal lobe dysfunction could be the most prominent [22]. Previous studies have reported that poor performance of attention, psychomotor, and executive function were associated with antidepressant treatment [32,33], while they were still controversial [34,35]. Therefore, cognitive function tests, including Finger-Tapping Test (FTT, represented as motor function), Continuous Performance Test (CPT, represented as attention), and Wisconsin Card Sorting Test (WCST, represented as executive function), were used in the models to predict the outcomes of antidepressants in the current study.

The FTT consists of tapping with the index finger on a computer mouse as many times as possible within 10 s. The test was repeated three consecutive times and performed randomly across subjects, and the order was kept constant for each subject at each session. The average number of taps was then calculated [36].

#### 2.4.2. Continuous Performance Test (CPT)

The CPT is a psychological test for humans that primarily measures attention [37,38]. The critical stimulus may be defined either as a particular single stimulus out of the available set (X task: subjects were asked to respond to the number “9”) or a particular sequence of two stimuli out of the available set (AX task: subjects were asked to respond whenever the number “9” was preceded by the number “1”). Only the AX task was used in the present study. Each test session began with 2 min of practice (repeated if necessary) to ensure that the subjects knew how to press the button correctly. During the test, numbers from 0 to 9 were randomly presented for 50 milliseconds each at a rate of one per second. Each subject underwent two sessions, including the nonmasked task and the 25% masked task. During the masked session, a pattern of snow was used to toggle the background and foreground so that the image was visually distorted. The masked CPT is more sensitive in detecting cognitive deficits. Subject responses were recorded automatically on a diskette using a CPT machine (Sunrise Systems, version 2.20, Pembroke, MA, USA) [39].

#### 2.4.3. Wisconsin Card Sorting Test (WCST)

We used a computerized version of the WCST conducted by an experienced clinical neuropsychologist. There were 64 cards in the test. All definitions of indices were as described in the WCST manual [40]. Subjects were required to match response cards to four stimuli along one of three dimensions (color, form, and number) based on verbal feedback (correct or wrong) that did not give any information about the dimensions. The index of the completed categories and preservative errors were used to assess the performance on the WCST [41,42].

### 2.5. Statistical Analysis

Categorical variables are expressed as numbers and percentages, and continuous variables are expressed as the means ± standard deviation (SD) unless otherwise specified. Categorical variables were assessed by using chi-square tests. Continuous variables were assessed by Student’s *t*-test. If a continuous variable was not normally distributed, the statistical analysis was assessed by the Mann–Whitney U test. The two-tailed level of significance was set at 0.05.

### 2.6. Machine Learning

#### 2.6.1. Data Preprocessing and Feature Selection

To eliminate any effect of different scales between variables, we standardized all continuous variables. Limited to our small sample size, we performed feature selection to address the problem of the “curse of dimensionality”. Univariate feature selection was applied with a generalized linear model, and sex and age were considered covariates to correct the main effect of each variable. Finally, we compared the performance of the machine learning model regardless of whether predictors were extracted.

#### 2.6.2. Feedforward Neural Network Model

**Figure 1:**
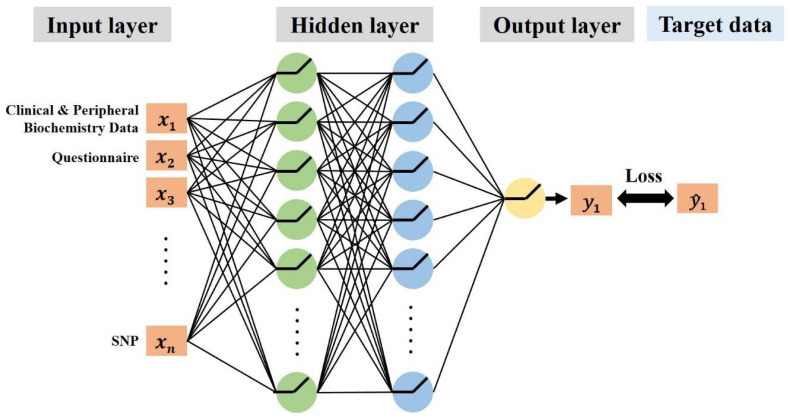
The architecture of the multilayer feedforward neural networks used to predict the treatment outcome.

**Figure 2:**
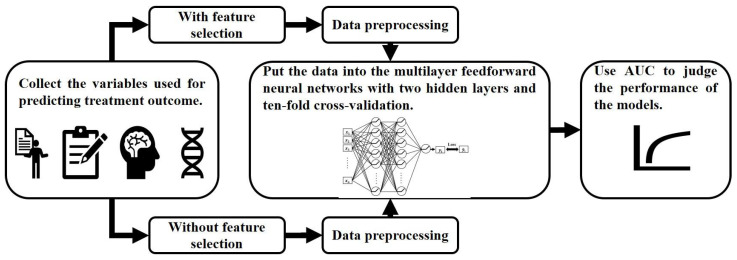
The procedure to construct multilayer feedforward neural networks for predicting the treatment outcome.

All participants were randomly separated into a training dataset (75% of participants) and an evaluation dataset (25% of participants). Then, a feedforward neural network with stratified tenfold cross-validation was applied to construct models predicting remission for fluoxetine or venlafaxine within the training dataset. The first layer of the neural network was the input layer, in which each unit received a one-dimensional data vector containing the features of the patient. Our models had two hidden layers, and the number of units was also set as one hyperparameter. The last layer was the output layer that performed the classification (Figure 1). To evaluate the performance of the models, the accuracy and areas under the receiver operating characteristic curves were assessed (Figure 2).

## 3. Results

### 3.1. Demographic Characteristics and Peripheral Biochemistry

We recruited 70 MDD patients who completed the 6-week antidepressant treatment. Among them, 25 patients achieved a remission (35.7%), and 45 patients did not (64.3%). No significant differences were observed between the groups in terms of demographics such as age, sex, and BMI (Table 1). MDD patients with remission had higher levels of oxytocin (35.9 ± 25.4 vs. 26.5 ± 11.7, *p* = 0.039) and cortisol (17.2 ± 6.4 vs. 13.0 ± 6.8, *p* = 0.011).

### 3.2. Questionnaire Score

We found that remission patients had higher scores on the overall (5.6 ± 1.3 vs. 4.5 ± 1.7, *p* = 0.015) and physical health (18.7 ± 3.8 vs. 15.6 ± 5.3, *p* = 0.005) domains of the WHOQoL (Table 2). In addition, remission patients also had higher scores in all domains (perceived crisis social support: 24.6 ± 4.6 vs. 20.6 ± 6.0, *p* = 0.026; received crisis social support: 30.3 ± 4.6 vs. 24.5 ± 7.7, *p* = 0.001; perceived routine social support: 23.4 ± 5.4 vs. 19.8 ± 6.6, *p* = 0.047; received routine social support: 26.5 ± 4.7 vs. 21.5 ± 6.5, *p* = 0.007) on the social support scale (Table 2). These results suggested that MDD patients with remission had a better quality of life and social support before receiving antidepressant treatment.

### 3.3. Cognitive Function

There was no significant difference in cognitive function performance between remission and nonremission patients (Table 3).

### 3.4. Genotype Frequencies of SNPs

Among the SNPs, the genotype frequencies of *OXTR* rs53576 polymorphisms were significantly different between remission and nonremission patients. There were more patients with the GG genotype (24.0% vs. 2.2%, *p* = 0.014) in the remission group (Table 4).

### 3.5. The Performance of the Feedforward Neural Network Model in Predicting the Remission of Patients

#### 3.5.1. Training Model without Feature Selection

Furthermore, we established prediction models using a single domain of categorical variables (Models 1, 2, 3, and 4), and the results demonstrated that using questionnaire scores as predictors had the highest AUC (0.770 ± 0.154) (Table 5). After permutation and combination of different domains of categorical variables, the AUC of Models 5, 8, 9, 11, 12, and 15 ranged between 0.7 and 0.8 (Table 5), which is acceptable discrimination [43].

#### 3.5.2. Training Model after Feature Selection

Moreover, to extract more precise variables that influenced treatment remission, we put those variables achieving significant differences (*p* < 0.05, from Table 1, Table 2, Table 3 and Table 4) into a feedforward neural network model for training. The training outcome is shown in Table 6. Models 1S, 2S, and 3S were established using a single domain of categorical variables, and the results demonstrated that using questionnaire scores as predictors had the highest AUC (0.763 ± 0.124) (Table 6). After permutation and combining the two different domains of the categorical variables, Models 4S, 5S, and 6S demonstrated similar or even better AUC performance than Models 1S to 3S. Furthermore, when we established a model using a combination of all different domains of the signature categorical variables, the highest AUC (0.825 ± 0.109) of prediction was obtained. In addition, we found that using filtered variables (those achieving significance differences) to establish the models demonstrated better performance than those without filtering (Table 5 and Table 6).

## 4. Discussion

Recent studies have reported drug efficacy prediction models for depression [17,18,19]. However, due to the complexity and heterogeneity of MDD and the difficulty of sample collection, they have not been able to come up with consistent conclusions. Here, we demonstrated that models developed with a deep neural network of deep learning to predict the treatment outcomes of antidepressants demonstrated clinical utility in drug-naïve and first-diagnosis MDD patients during the severe depressive stage. Additionally, we maximized the prediction accuracy of the treatment outcomes of antidepressants among MDD patients using combinations of different domains of signature profiles, including clinical features, peripheral biochemistry, psychosocial factors, and genetic variants, and the prediction was obtained with a good AUC range of 0.75 to 0.83. Therefore, deep neural network models of deep learning demonstrated promise for predicting the complexity of treatment outcomes, such as antidepressants. Additional validation of the model with an external database is necessary to confirm its generalization ability. Additional experiments are required to optimize the prediction rate and develop new model calculation methods, such as generative adversarial networks. From our pilot study, it is expected that prediction models of drug efficacy can be applied in clinical practice to achieve the goal of precise, individualized medicine.

### 4.1. Oxytocin and Cortisol

MDD is a complex and highly heterogeneous disorder whose pathophysiology and mechanisms of pharmacotherapy are not fully understood. The relationship between depression and hypothalamic–pituitary–adrenal axis (HPA axis) dysregulation has been the most widely discussed. Approximately 60% of patients with major depressive disorder have an increase in the activity of the HPA axis [44]. A sustained increase in HPA axis activity is also thought to be associated with a resistance to antidepressant medication [45]. Oxytocin is secreted by neurons in the supraoptic nuclei and paraventricular nuclei of the hypothalamus and plays an important role in production, parenting, and social bonding. In addition, it has also been pointed out that oxytocin has an effect of reducing anxiety and stress, and one possible mechanism is by reducing the activity of the HPA axis, but the precise mechanism has not been established [46]. In a mouse model, intra raphe infusion of oxytocin resulted in increased release of serotonin from the median raphe nucleus, suggesting an interaction between the oxytocin and serotonin systems that is possibly related to the therapeutic effect of SSRIs. This may also underlie their anxiolytic effects [47]. According to the results of our study, the plasma oxytocin level of the remission patients before treatment was significantly higher than that of the nonremission patients, which is consistent with the possible mechanism mentioned above. However, although the plasma oxytocin level could be correlated with the brain oxytocin level in previous reports, more research is necessary to investigate the role of oxytocin in the mechanism of antidepressant treatment response in MDD patients.

In MDD patients, it has been observed that a continuous increase in the activity of the HPA axis may be due to the abnormal signal transduction of glucocorticoid receptors or the dysregulation of corticotrophin releasing hormone nerves [48]. Therefore, the relationship between cortisol and MDD has been studied. Jain, FA et al. suggested that the efficacy of antidepressants was related to the interaction between the blood cortisol level and age. Taking early or middle adulthood as the cutoff point, for patients younger than the cutoff point, the lower the blood cortisol level is, the better the therapeutic effect. In patients older than the cutoff point, the lower the blood cortisol level is, the worse the treatment effect [49]. However, there was a controversial report stating that it is not appropriate to directly predict the efficacy of antidepressant drugs based on the level of cortisol. The degree of change in the response of the HPA axis to external stress stimuli should be used as a judgment factor for antidepressant efficacy prediction [48]. Whether cortisol levels can be used as a predictor of antidepressant efficacy needs to be confirmed.

### 4.2. Social Support Scale and Quality of Life

Recent studies have pointed out that environmental stress, the inflammatory response, and the occurrence of MDD are closely related to the prognosis of the disease, which prompted the proposal of the social signal transduction theory of depression [50]. This theory states that environmental stress, such as social threats, social rejection, and interpersonal loss, affects the anterior insula and dorsal anterior cingulate cortex, and through the sympathetic nervous system or HPA axis, it modulates the expression of peripheral immune cell genes and induces cells to release proinflammatory cytokines such as interleukin-6, interleukin-1β, tumor necrosis factor-α, and C-reactive protein [51]. Thus, these abnormally increased inflammatory factors return to the brain through the circumventricular organ or the vagus nerve, affecting cognitive function, behavior, and mood and leading to depression. Depending on the severity of the inflammation, it can further affect the efficacy of antidepressants [52].

In addition, lower perceived social support scores are associated with a worse prognosis in depression [53]. The mechanism of perceived social support acting on the human body may be related to the oxytocin system. Reducing the activity of the HPA axis may allow depression to have a better prognosis and treatment outcome [54]. According to our study, the social support scale scores of remission patients at baseline were significantly higher than those of nonremission patients and demonstrated excellent performance in building machine learning models for predicting the treatment outcomes of antidepressants.

Promoting and intervening in the quality of life (QoL) of patients with mental disorders has increasingly become an important goal of clinicians [55]. According to the literature, patients with MDD have worse QoL than those without depression tendencies and those with other chronic diseases. In addition, patients with MDD with poor QoL may be at risk of relapse after treatment [56,57]. Based on our data, nonremission patients had a lower QoL in the domain of physical health before receiving antidepressant treatment. Although the biological mechanism by which QoL can predict the outcomes of antidepressants is not yet clear, our results provide further support to QoL influencing the status of MDD and its related treatment outcomes.

### 4.3. OXTR and Treatment Response

The oxytocin receptor gene (*OXTR*) has been found to be associated with neuropsychiatric diseases [58], among which the rs53576 locus selected to be investigated in this study has many related reports [59]. The *OXTR* variant rs53576 is located on human chromosome 3, its ancestral allele is guanine, and the minor allele is adenine. Individuals carrying the A allele tend to exhibit socioemotional development deficits [60]. One study found that women who are rs53576 AA homozygotes have increased harm avoidance relative to G carriers. In addition, there are also differences in the brain structure, such as a smaller amygdala volume and a reduced resting-state functional coupling between the prefrontal cortex and amygdala, which also means greater susceptibility to stress. The rs53576 genotype is also related to social support. Individuals with the G allele can obtain protective effects from social support, and this result may be due to the lower cortisol response to stress [61]. The cortisol response may also affect the response to drug treatment.

The results of our study demonstrated that the proportion of nonremission patients with the GG genotype was lower than that of remission patients, and the proportion of nonremission patients with the AA genotype was higher than that of remission patients, which is consistent with the above discussion. Our outcome may further provide insight into the relationship between the rs53576 polymorphism and drug efficacy. In addition, when profiles of the genetic variants and the other types of variables were considered together, the predictive ability of our models had an upward trend (AUC increased). The reason could be that there were interactions or synergic effects between different domains of the categorical variables, including clinical features, peripheral biochemistry, scores on the questionnaire, and genetic variants.

### 4.4. Antidepressant Treatment Response Prediction Model

Due to the complexity and heterogeneity of mood disorders, the treatment response is difficult to predict before the patients try a medication [62]. Studies on the prediction of antidepressant treatment outcomes have built powerful models from different points of view and information, such as using pharmacogenomics (single nucleotide polymorphisms), social environmental factors, clinical indices, and brain imaging [15,63]. However, due to inconsistencies in the way the experiments are conducted or the methods used for evaluating the results, it is difficult to obtain a consistent view of the research results of different types of information, and further integration is also difficult. Therefore, machine learning has been introduced because of its ability to integrate different types of data for deep data mining, and the current widely used method is a branch of machine learning—deep learning, which also has the advantage of being able to apply many methods to avoid overfitting [64,65,66,67,68].

The extraction/selection of appropriate data as the input for the neural network is a very important issue; otherwise, it will affect the prediction results and stability [69]. Too many parameters may cause the model to overfit, and it may also cause variables with a high correlation to have an effect of repeated calculation and make the weights of the neural network have a bias to improve the explanatory and predictive power of certain variables, also called multicollinearity [70]. The current data extraction/selection methods are mainly divided into two categories: (1) the first is through a literature review; (2) the second is through a preliminary analysis of the data, setting thresholds for significant differences, and then selecting variables. In addition, many studies reduced the dimensionality of the dataset before performing variable extraction, such as principal component analysis, to avoid overfitting caused by too many parameters [71]. Our current study, using the second type of method and normalized variables as inputs, demonstrated the good performance of the prediction model.

### 4.5. Limitation of the Study

Nevertheless, our findings need to be interpreted in terms of some limitations. First, there was a small sample size and a short duration of the antidepressant treatment. Although the larger sample size is needed to construct a predictive model, there are some precedents in which the scale of sample is below one hundred [20]. To overcome the limitation of a small sample size, analytical methods can be applied such as feature selection [72] and dimensionality reduction [73]. In our study, we extracted significant features to establish predictive models which demonstrated the good performance. We would like to expand the scale in further study. Second, nonmedication factors that may have confounded the results of the study, such as diet, alcohol, exercise, and comorbidities, were not accounted for, although we have carefully collected the psychosocial factors, indices of peripheral biochemistry, and genetic variants. Third, MDD patients using different drugs were not subgrouped to perform these analyses due to the small sample size. It would be worthwhile to investigate medication effects, as there may be distinct factors affecting the efficacy of different classes of antidepressants. The inclusion of different types of drugs to construct a prediction model is required in the future to fulfill the demand in the real world. In addition, all of the MDD patients in the current study were drug naïve, first diagnosed and at a severe depressive stage, and the prediction model could not be applied to recurrent or treatment-resistant MDD patients. Fourth, our prediction model needs to be validated and confirmed as to its generalization ability through an external dataset, although the current model has demonstrated good accuracy for predicting the treatment outcomes of antidepressants. Fifth, increasing SNPs’ coverage on each gene is better for further understanding the role of genetic factors in the therapeutic action of antidepressants. Finally, further prospective studies might provide solid evidence for the concerns raised in the current study. The deep neural network used in the current study additionally provided us with the interactivity of predictor variables to obtain better prediction but limits our understanding of how each variable interacts with others, which requires further mechanistic studies.

## 5. Conclusions

In conclusion, our study integrated different domains of categorical variables, including clinical features, peripheral biochemistry, scores on questionnaires, and genetic variants, to establish multiple models and explore their predictive ability for antidepressant treatment outcomes of MDD patients. The results suggested that a combination of the extraction of clinical features, peripheral biochemistry, psychosocial factors, and genetic variants demonstrated good performance for outcome prediction. Therefore, this complex interactions model, developed through a deep neural network, could be useful at the clinical level for predicting individualized outcomes of antidepressants. Additional clinical studies are necessary to validate the accuracy of the predictions.

## Figures and Tables

**Table 1:** Demographic characteristics and peripheral biochemistry of the remission and nonremission patients.

Characteristics	Remission	Nonremission	Comparison	
	(N = 25)	(N = 45)		
	Mean ± SD	Mean ± SD	t/U/χ^2^	*p*
Clinical features				
Age, years	40.8 ± 15.6	39.1 ± 12.0	536.5	0.754
Gender, male (%)	36.0%	22.2%	0.925	0.336
HDRS scores of baseline	22.9 ± 5.4	24.5 ± 5.6	1.184	0.242
Peripheral biochemistry				
BH, cm	161.1 ± 7.2	160.2 ± 8.0	420.5	0.360
BW, kg	58.98 ± 11.56	55.33 ± 11.99	402.0	0.242
BMI, kg/m^2^	22.69 ± 3.95	21.48 ± 3.75	−1.226	0.226
SBP, mmHg	117.6 ± 19.2	113.2 ± 16.8	−0.952	0.346
DBP, mmHg	76.4 ± 9.6	74.2 ± 9.7	−0.921	0.361
Sugar profiles				
AC sugar, mg/dL	96.1 ± 12.7	92.2 ± 11.7	428.5	0.204
Insulin, µIn/mL	6.77 ± 6.97	7.87 ± 9.44	611.5	0.446
HbA1c (%)	5.67 ± 0.44	5.56 ± 0.30	442.0	0.398
HOMA-IR	1.72 ± 1.90	1.95 ± 2.72	549.0	0.794
HOMA-β (%)	76.83 ± 69.15	88.79 ± 68.51	615.0	0.269
Lipid profiles				
Cholesterol, mg/dL	202.88 ± 45.84	189.02 ± 40.78	416.5	0.154
TG, mg/dL	113.83 ± 65.07	103.77 ± 70.81	458.5	0.376
HDL, mg/dL	56.57 ± 13.54	55.55 ± 14.05	505.5	1.000
LDL, mg/dL	126.35 ± 41.94	112.27 ± 38.83	413.5	0.224
LDL/HDL	2.32 ± 0.86	2.19 ± 1.07	423.0	0.276
Other biochemical indices				
C-peptide, ng/mL	1.98 ± 1.30	1.92 ± 1.83	484.5	0.416
Cortisol, µg/dL	17.2 ± 6.4	13.0 ± 6.8	348.0	0.011 *
Leptin, ng/mL	8.78 ± 6.89	10.88 ± 14.36	480.0	0.388
Oxytocin, pg/mL	35.9 ± 25.4	26.5 ± 11.7	448.0	0.039 *
hsCRP, pg/mL	287,440.0 ± 311,763.7	261,172.3 ± 357,027.6	511.0	0.721

Abbreviations: SD: standard deviation, HDRS: Hamilton Depression Rating Scale, BH: body height, BW: body weight, BMI: body mass index, SBP: systolic blood pressure, DBP: diastolic blood pressure, HOMA-IR: homeostasis model assessment-estimated insulin resistance, HOMA-β: homeostasis model assessment for pancreatic β cell function, TG: triglyceride, HDL: high-density lipoprotein, LDL: low-density lipoprotein, and hsCRP: high sensitive C-reactive protein. * *p* < 0.05.

**Table 2:** Questionnaire scores of the remission and nonremission patients.

Questionnaire	Remission	Nonremission	Comparison	
	(N = 25)	(N = 45)		
	Mean ± SD	Mean ± SD	t/U	*p*
WHOQoL				
Overall	5.6 ± 1.3	4.5 ± 1.7	238.5	0.015 *
Physical health	18.7 ± 3.8	15.6 ± 5.3	213.5	0.005 *
Psychological	15.0 ± 3.2	14.0 ± 4.1	−0.909	0.368
Social relationship	13.6 ± 3.5	12.4 ± 3.6	308.0	0.207
Environment	34.1 ± 6.3	31.1 ± 5.8	−1.857	0.070
Social support scale				
Perceived crisis social support	24.6 ± 4.6	20.6 ± 6.0	196.0	0.026 *
Received crisis social support	30.3 ± 4.6	24.5 ± 7.7	−3.476	0.001 *
Perceived routine social support	23.4 ± 5.4	19.8 ± 6.6	229.0	0.047 *
Received routine social support	26.5 ± 4.7	21.5 ± 6.5	190.5	0.007 *
Life event score				
Total score	9.5 ± 8.0	10.6 ± 10.6	329.5	1.000

Abbreviations: SD: standard deviation and WHOQoL: the World Health Organization quality of life. * *p* < 0.05.

**Table 3:** Cognitive function of the remission and nonremission patients.

Cognitive Function	Remission	Nonremission	Comparison	
	(N = 25)	(N = 45)		
	Mean ± SD	Mean ± SD	U	*p*
Finger-Tapping Test				
Dominant finger	38.4 ± 11.1	36.8 ± 11.4	412.5	0.584
Nondominant finger	38.0 ± 11.2	35.5 ± 8.2	398.0	0.364
Wisconsin Card-Sorting Test				
Perseverative errors	18.8 ± 14.5	16.0 ± 12.6	436.0	0.718
Completed categories	1.3 ± 1.6	1.9 ± 1.6	568.0	0.125
Continuous Performance test				
Unmasked	3.83 ± 1.38	3.73 ± 1.08	367.0	0.226
Masked	3.07 ± 1.46	2.74 ± 1.26	328.0	0.264

Abbreviations: SD: standard deviation.

**Table 4:** Genotype of the remission and nonremission patients.

SNP	Related Gene	Chromosome	Reference Allele	Remission			Non-Remission			Comparison
				(N = 25)			(N = 45)			
				%			%			*p*
rs6265	BDNF	11	C	CC	CT	TT	CC	CT	TT	0.772
				40.0	32.0	28.0	37.8	40.0	22.2	
rs5443	GNB3	12	C	CC	CT	TT	CC	CT	TT	0.459
				28.0	40.0	32.00	15.6	46.7	37.7	
rs6313	HTR2A	13	G	AA	AG	GG	AA	AG	GG	0.949
				32.0	48.0	20.0	35.6	44.4	20.0	
rs6295	HTR1A	5	G	CC	CG	GG	CC	CG	GG	0.828
				8.0	36.0	56.0	8.9	28.9	62.2	
rs16944	IL1B	2	A	AA	AG	GG	AA	AG	GG	0.446
				12.0	36.0	52.0	24.4	33.3	42.3	
rs1800532	TPH1	11	G	TT	GT	GG	TT	GT	GG	0.143
				36.0	36.0	28.0	15.6	51.1	33.3	
rs25533	SLC6A4	17	A	AA	AG	GG	AA	AG	GG	0.302
				76.0	24.0	0.0	71.1	20.0	8.9	
rs53576	OXTR	3	G	AA	AG	GG	AA	AG	GG	0.014 *
				40.0	36.0	24.0	53.3	44.4	2.3	

Abbreviation: SNP: single nucleotide polymorphism. * *p* < 0.05.

**Table 5:** The results of each model with a combination of full data from different domains for predicting the treatment outcome (remission or nonremission) using multilayer feedforward neural networks with two hidden layers.

Model (No.)	Number of Markers	Accuracy (Mean ± SD)	AUC (Mean ± SD)
Age, sex, HDRS, clinical and peripheral biochemistry (1)	23	64.286 ± 7.143%	0.690 ± 0.281
Age, sex, HDRS, questionnaire (2)	15	64.286 ± 7.143%	0.770 ± 0.154
Age, sex, HDRS, cognitive function (3)	9	64.286 ± 7.143%	0.700 ± 0.152
Age, sex, HDRS, SNP (4)	11	65.714 ± 6.999%	0.612 ± 0.177
Age, sex, HDRS, clinical and peripheral biochemistry, questionnaire (5)	35	70.000 ± 10.000%	0.722 ± 0.160
Age, sex, HDRS, clinical and peripheral biochemistry, cognitive function (6)	29	65.714 ± 9.476%	0.698 ± 0.238
Age, sex, HDRS, clinical and peripheral biochemistry, SNP (7)	31	62.857 ± 6.999%	0.650 ± 0.203
Age, sex, HDRS, questionnaire, cognitive function (8)	21	64.286 ± 7.143%	0.762 ± 0.184
Age, sex, HDRS, questionnaire, SNP (9)	23	67.143 ± 11.158%	0.717 ± 0.123
Age, sex, HDRS, cognitive function, SNP (10)	17	64.286 ± 9.583%	0.662 ± 0.188
Age, sex, HDRS, clinical and peripheral biochemistry, questionnaire, cognitive function (11)	41	67.143 ± 9.147%	0.737 ± 0.232
Age, sex, HDRS, questionnaire, cognitive function, SNP (12)	29	65.714 ± 13.093%	0.720 ± 0.195
Age, sex, HDRS, clinical and peripheral biochemistry, cognitive function, SNP (13)	37	70.000 ± 10.000%	0.633 ± 0.243
Age, sex, HDRS, clinical and peripheral biochemistry, questionnaire, SNP (14)	43	67.143 ± 12.857%	0.692 ± 0.163
Age, sex, HDRS, clinical and peripheral biochemistry, questionnaire, cognitive function, SNP (15)	49	68.571 ± 10.690%	0.753 ± 0.154

Clinical and peripheral biochemistry: including all variables in Table 1. Questionnaire: including all variables in Table 2. Cognitive function: including all variables in Table 3. SNP: single nucleotide polymorphism, including all variables in Table 4. Abbreviation: HDRS: 17-item Hamilton depression rating scale at baseline. AUC: the area under the receiver operating characteristic curve. SD: standard deviation.

**Table 6:** The result of each model with a combination of selected data from different domains for predicting the treatment outcome (remission or nonremission) using multilayer feedforward neural networks with two hidden layers.

Model (No.)	Number of Markers	Accuracy (Mean ± SD)	AUC (Mean ± SD)
Age, sex, HDRS, clinical and peripheral biochemistry (1S)	4	64.286 ± 7.143%	0.707 ± 0.201
Age, sex, HDRS, questionnaire (2S)	11	62.857 ± 6.998%	0.763 ± 0.124
Age, sex, HDRS, SNP (3S)	4	64.286 ± 7.143%	0.757 ± 0.199
Age, sex, HDRS, clinical and peripheral biochemistry, questionnaire (4S)	13	64.286 ± 7.143%	0.815 ± 0.184
Age, sex, HDRS, clinical and peripheral biochemistry, SNP (5S)	5	67.143 ± 9.147%	0.763 ± 0.196
Age, sex, HDRS, questionnaire, SNP (6S)	12	65.714 ± 11.429%	0.815 ± 0.137
Age, sex, HDRS, clinical and peripheral biochemistry, questionnaire, SNP (7S)	13	68.571 ± 12.454%	0.825 ± 0.109

Clinical and peripheral biochemistry: including levels of cortisol and oxytocin. Questionnaire: including overall domain and physical health domain of WHOQoL and all domains of the social support scale. SNP: single nucleotide polymorphism, including rs53576 (*OXTR*). Abbreviation: HDRS: 17-item Hamilton depression rating scale at baseline. AUC: the area under the receiver operating characteristic curve. SD: standard deviation.

## Data Availability

The data that support the findings of this study are available from the National Cheng Kung University. Restrictions apply to the availability of these data, which were used under license for this study. Data are available Hui Hua Chang with the permission of National Cheng Kung University.

## Supplementary Materials

The following supporting information can be downloaded at: https://www.mdpi.com/article/10.3390/jpm12050693/s1, Figure S1: Flowchart for MDD patients in the study; Table S1: Demographic characteristics and peripheral biochemistry of the enrolled and not enrolled patients; Table S2: Questionnaire scores of the enrolled and not enrolled patients; Table S3: Cognitive function of the enrolled and not enrolled patients.
