# Supplementary material for: Predicting the Treatment Outcomes of Antidepressants Using a Deep Neural Network of Deep Learning in Drug-Naïve Major Depressive Patients
Source: J Pers Med. 2022 Apr 26;12(5):693. doi: 10.3390/jpm12050693 (PMC9146151; doi:10.3390/jpm12050693)
Supplement: Supplementary file 1 [file jpm-12-00693-s001.zip › jpm-1681862-supplementary.pdf]

Supplementary Table S1. Demographic characteristics and peripheral biochemistry of the enrolled and not enrolled patients.

Characteristics	Enrolled (N=70)	Not enrolled (N=42)	Comparison	
	Mean ± SD	Mean ± SD	t/U/χ^2	*p*
Clinical features				
Age, years	39.7 ± 13.3	39 ± 11.8	2059.5	0.729
HDRS scores of baseline	23.9 ± 5.6	24.4 ± 5.6	0.474	0.637
Peripheral biochemistry				
BH, cm	160.6 ± 7.6	161.1 ± 7.1	1357.5	0.606
BW, kg	56.76 ± 11.87	58.43 ± 11.94	1368.0	0.416
BMI, kg/m^2	21.95 ± 3.84	22.48 ± 4.42	0.617	0.539
SBP, mmHg	114.9 ± 17.8	113 ± 16.2	-0.578	0.565
DBP, mmHg	75.1 ± 9.6	76.6 ± 12.4	0.667	0.507
Sugar profiles				
AC sugar, mg/dl	93.6 ± 12.1	91.1 ± 12.3	1234.0	0.424
Insulin, μIn/ml	7.47 ± 8.59	6.38 ± 5.4	1315.5	0.850
HbA1c (%)	5.60 ± 0.36	5.62 ± 0.46	1245.5	0.690
HOMA-IR	1.87 ± 2.45	1.54 ± 1.58	1284.0	0.788
HOMA-β (%)	84.57 ± 68.46	80.85 ± 47.59	1353.5	0.861
Lipid profiles				
Cholesterol, mg/dL	193.91 ± 42.81	190.08 ± 46.94	1269.5	0.567
TG, mg/dL	107.32 ± 68.52	100.43 ± 66.04	1294.5	0.679
HDL, mg/dL	55.90 ± 13.78	60.97 ± 15.51	1.693	0.095
LDL, mg/dL	117.10 ± 40.18	111.49 ± 44.10	1164.5	0.354
LDL/HDL	2.23 ± 1.00	1.98 ± 0.95	1107.5	0.193
Other biochemical indices				
C-peptide, ng/ml	1.94 ± 1.65	2.06 ± 1.75	1323.5	0.890
Cortisol, μg/dL	14.5 ± 6.9	13.3 ± 4.2	1221.0	0.427
Leptin, ng/mL	10.12 ± 12.18	11.02 ± 13.25	1373.5	0.687
Oxytocin, pg/mL	29.9 ± 18.3	36.1 ± 18.1	1274.0	0.055
hsCRP, pg/mL	270308.9 ± 339873.3	335136.7 ± 455223.1	1428.0	0.600

Abbreviations: SD: standard deviation, HDRS: Hamilton Depression Rating Scale, BH: body height, BW: body weight, BMI: body mass index, SBP: systolic blood pressure, DBP: diastolic blood pressure, HOMA-IR: homeostasis model assessment-estimated insulin resistance, HOMA-β: homeostasis model assessment for pancreatic β cell function, TG: triglyceride, HDL: high-density lipoprotein, LDL: low-density lipoprotein, hsCRP: high sensitive C-reactive protein.

Supplementary Table S2. Questionnaire scores of the enrolled and not enrolled patients.

Questionnaire	Enrolled (N=70)	Not enrolled (N=42)	Comparison	
	Mean ± SD	Mean ± SD	t/U	p
WHOQoL				
Overall	4.9 ± 1.6	5.1 ± 1.8	1017.0	0.692
Physical health	16.8 ± 5.0	19.1 ± 5.2	1.982	0.054
Psychological	14.4 ± 3.8	14 ± 4.5	-0.345	0.731
Social relationship	12.9 ± 3.5	13.4 ± 3.2	0.668	0.506
Environment	32.2 ± 6.1	32.7 ± 6.6	0.322	0.748
Social support scale				
Perceived crisis social support	22.1 ± 5.8	23.1 ± 5.4	952.0	0.398
Received crisis social support	26.6 ± 7.3	28.5 ± 6.0	1.285	0.203
Perceived routine social support	21.1 ± 6.3	21.2 ± 5.6	878.5	0.900
Received routine social support	23.4 ± 6.4	25.6 ± 7.7	989.0	0.166
Life event score				
Total score	10.2 ± 9.5	13.5 ± 15.3	844.5	0.536

Abbreviations: SD: standard deviation, WHOQoL: the World Health Organization quality of life.

Supplementary Table S3. Cognitive function of the enrolled and not enrolled patients.

Cognitive Function	Enrolled (N=70)	Not enrolled (N=42)	Comparison	
	Mean ± SD	Mean ± SD	U	*p*
Finger-Tapping Test				
Dominant finger	37.4 ± 11.2	37.4 ± 11.5	1226.5	0.992
Nondominant finger	36.4 ± 9.3	35.3 ± 9.2	1197.0	0.731
Wisconsin Card-Sorting Test				
Perseverative errors	17 ± 13.2	17.4 ± 12.4	1391.0	0.460
Completed categories	1.7 ± 1.6	1.6 ± 1.5	1252.0	0.850
Continuous Performance test				
Unmasked	3.77 ± 1.18	3.75 ± 1.07	1285.0	0.968
Masked	2.86 ± 1.33	2.98 ± 1.12	1156.5	0.796

Abbreviations: SD: standard deviation.

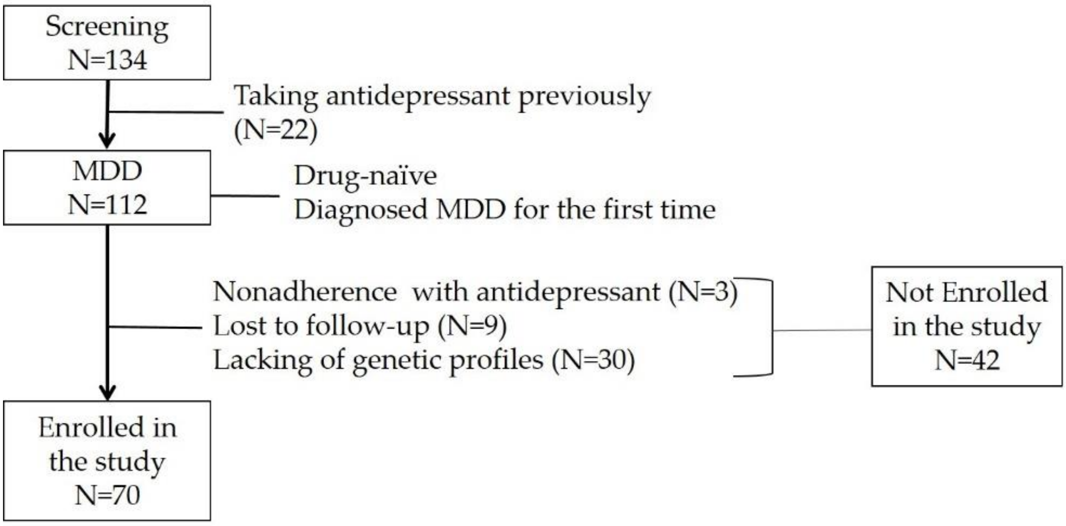

Supplementary Figure S1. Flowchart for MDD patients in the study.
